# The effects of counseling via a smartphone application on microentrepreneurs’ work ability and work recovery: a study protocol

**DOI:** 10.1186/s12889-020-8449-7

**Published:** 2020-04-03

**Authors:** J. Laitinen, E. Korkiakangas, J. P. Mäkiniemi, S. Tiitinen, P. Tikka, H. Oinas-Kukkonen, A. M. Simunaniemi, S. Ahola, J. Jaako, M. Kekkonen, M. Muhos, K. Heikkilä-Tammi, H. Hannonen, S. Lusa, A. Punakallio, J. Oksa, S. Mänttäri, S. Ilomäki, A. Logren, J. Verbeek, J. Ruotsalainen, J. Remes, J. Ruusuvuori, T. Oksanen

**Affiliations:** 1 Finnish Institute of Occupational Health, Helsinki, Finland; 2 Faculty of Management and Business, Tampere University, Tampere, Finland; 3 Faculty of Sciences, Tampere University, Tampere, Finland; 4 Faculty of Information Technology and Electrical Engineering, Oulu Advanced Research on Service and Information Systems (OASIS), University of Oulu, Oulu, Finland; 5 Kerttu Saalasti Institute, University of Oulu, Oulu, Finland

## Background

The sustained work ability of microentrepreneurs has a significant impact on regional labor markets and society in general, because microenterprises form a significant portion of all enterprises in the EU and in Finland. In such enterprises, with 1–9 employees and whose annual turnover and/or annual balance sheet total does not exceed two million euros [1], working long hours and finding a healthy work-life balance tends to be challenging: as many as 55% of the self-employed with employees and 35% of those without employees reported working 48 h or more per week [2]. Heavy workloads, financial insecurity, and lack of free time are known to lead to increased levels of stress [3]. Health concerns following such challenges include cardiovascular diseases [4, 5], coronary heart disease, stroke and type 2 diabetes [6, 7]. Thus, the promotion of work ability highlights the importance of recovery from work [8].

A few studies have been published on the factors associated with work ability among microentrepreneurs [9, 10]. Cross-sectional studies have found good planning and control over work; flexibility at work; good social support from family, friends and other entrepreneurs; and regular exercise to be associated with maintaining entrepreneurs’ good health [11]. Furthermore, intervention studies have produced promising results related to increasing physical activity [12] and reducing alcohol consumption [13]. There are studies that address mental health apps, for example stress management intervention [14]. There are also some studies that address recovery as primary outcome among employees who suffer from both work-related strain and sleep problems [15]. However, to our knowledge, there is a gap of intervention studies on the promotion of microentrepreneurs’ work ability and recovery from work using a smartphone application as the key means of delivering counseling.

One reason for poor results from intervention studies might be that behavior change interventions often lack a solid theoretical background [16, 17], although some of the previous studies suggest a link between the application of a theory and the effectiveness of an intervention [18–20]. We adopted Self-Determination Theory as the theoretical framework for the intervention study as it has shown potential for effectiveness in meta-analyses and systematic reviews [21, 22].

SDT is a theory of human motivation and personality [23], which sees an individual as inherently active, motivated and having a tendency toward growth development. According to the SDT, an individual’s motivation is divided into amotivation, controlled motivation and autonomous or self-determined motivation. The more autonomous the motivation for behavior change is, the more likely the person is to change their current behavior and maintain the newly acquired behavior. Further, the theory sees three basic human needs as the driving force for developing more autonomous motivation. These are: 1) the need for autonomy (i.e. the feeling of being the origin of one’s own actions and possessing the ability to express one’s own free will through behavior), 2) the need for competence (i.e. the feeling of being a true actor, who is able to set goals and exploit their own skills and competences) and 3) the need for relatedness (i.e. being understood and appreciated) [23]. These general principles were chosen to be followed in designing the tasks for the application.

Previous systematic reviews of health behavior change as in our interventions have highlighted the effectiveness of some behavior change techniques over others. These include goal-setting [20, 24, 25], social support [26–28], social comparison [20, 29, 30] and giving information and instructions [30–32]. These techniques are applied in a way that fits with the guiding principles of SDT.

When aiming to utilize the evidence of effective behavior change techniques provided by previous systematic reviews, two points should be taken into account. First, systematic reviews [20, 25, 32] also offer inconsistent results in comparison to each other, which illustrates the importance of understanding not only what is done but also how the techniques are put into practice Secondly, some behavior change techniques have been utilized in previous interventions so infrequently that there is insufficient data to conduct meta-analysis and establish the bottom line regarding effectiveness [20, 30, 33]. One plausible explanation for this is publication bias. We published recently a reflective discussion on how we identified evidence-based behavior change techniques and counseling themes for the intervention [34].

Persuasive technology is increasingly used for promoting behavior change. It has been used in mobile applications for assisting people with issues such as weight management, sleep problems, and depression [35–37]. However, a recent review identified only a small number of currently available stand-alone apps that have been evaluated in RCTs, and concluded that overall, the evidence of effectiveness is of relatively low quality [38]. A recent conceptualization for developing applications with evidence-based effectiveness is known as the Behaviour Change Support Systems (BCSS) framework [39]. The BCSS framework aims to support and enable people in their behavior change efforts. Furthermore, it builds upon the Persuasive Systems Design model, developed for designing and evaluating information systems that aim to influence the attitudes or behaviors of users through technological persuasion without deception or coercion [40]. The intervention build by us utilizes these established and validated design and development approaches to implement carefully tailored intervention content into a persuasive smartphone application.

**Fig. 1:**
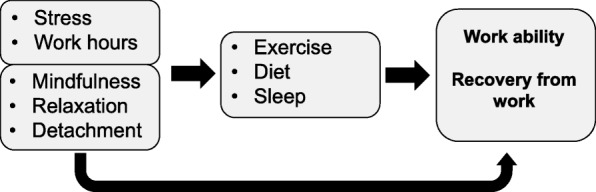
The basic intervention components of the intervention to enhance work ability (primary outcome) and recovery from work (secondary outcome) among microentrepreneurs

This paper describes the protocol of a randomized controlled trial, because publication of study protocols increases research quality and transparency [41]. The overall aim of the trial is to examine the effects of counseling delivered via a mobile application on work ability among microentrepreneurs. The secondary outcome will be change in recovery from work. In promotion of work ability, enhancing recovery from work is especially important among microentrepreneurs, who do long working hours weekly, and might have high levels of stress due to their personal responsibility of their business. Our hypothesis is that high level of stress and long working hours increase the need for recovery from work and decrease work ability independently and via unhealthy behaviors (Fig. 1). Mindfulness, relaxation and detachment from work improve recovery from work and work ability independently, and also through healthy behaviors. Healthy exercise, dietary and sleep habits enhance recovery from work independently as well as improved recovery from work improves work ability. When the levels of stress decrease, the need for recovery from work is lower, and moderate weekly amounts of working hours allow enough time for healthy behaviors in order to enhance physiological recovery of the body, that means repletion of body energy sources [42]. These basic intervention components (Fig. 1) have also direct effects on work ability. Additionally, the best ways to enhance recovery from work are different among those with low and high level of physical strenuousness of work, and thus the counselling will be tailored according to the level of physical strenuousness of work. Process evaluation will be conducted to detect the mechanisms of change and to study why the results are what they are.

## Methods

### Setting, participants and eligibility criteria

The design is a randomized controlled trial comparing one intervention arm (Recovery! application intervention) with a waiting list control group among microentrepreneurs in Finland. The waiting list control group will receive the Recovery! app six months from the commencement of the trial and will use it for eight weeks.

Eligible participants are Finnish-speaking microentrepreneurs (those with enterprises of 1–9 employees) whose companies are in Finland. The target group excludes the employees of these enterprises. Included participants must work full-time as entrepreneurs, understand Finnish, and have an Android phone. Entrepreneurs who are also employed in another organization, or who are on sick leave or off work for another reason, will be excluded.

### Intervention

The intervention consists of a mobile application for microentrepreneurs. The application aims to promote health and work-related behavior changes and to enhance recovery from work and work ability during working hours and free time.

The intervention group will receive the mobile application after baseline survey and will be asked to use it for at least eight weeks, but may continue longer. In the mobile app users select relevant modules (key components), goals for themselves, and subsequently select exercises to complete with guidance from the app. The users can also use additional support tools, such as a meal planner or step counter, and use tracking tools such as self-assessment of stress levels. These modules and tools will be described in closer detail in the forthcoming paper related to process evaluation. During the trial period the control group receives no intervention. However, the waitlist control group will receive the same mobile application at six months, and will be followed for two months.

#### Basic intervention components

The intervention program to promote work ability and recovery from work among microentrepreneurs will be delivered and implemented via a smartphone application), that is, it is designed directly as mobile application using compiled language [43], rather than web application to be used via mobile devices. The application was developed for the Android smartphone platform and it only works on smartphones using this operating system. The plan of action merges: 1) the contents based on the needs of the target population and evidence from research on health- and work-related behavior that enhances recovery from work, 2) a theoretical framework for counseling (Self Determination Theory, SDT), and counseling methods including behavior change techniques [44, 45], and 3) tailored content and counselling according to the trans-theoretical change model [46] and the physically strenuousness of the work.

The key contents (Fig. 1) and components of the mobile application to enhance work ability and recovery from work are 1) not too much stress, 2) effective working hours, 3) detachment from work, 4) relaxing), 5) sufficient, good-quality sleep, 6) regular meals that provide rhythm and energy for work, and 7) physical activity as the key to recovery for those doing physically strenuous work, and sitting less, moving more for those doing light, sedentary work. The application also includes short 8) mindfulness exercises for enhancing recovery and helping the user pay attention and focus better on situations at hand. These exercises are included because research shows that mindfulness may support behavior change (e.g. [47–49]) and promote recovery from work [49].

The behavior change techniques used in the application have been selected according to the evidence of their effectiveness. The selection of techniques also reflects the aims to 1) include various different techniques, as the diversity of techniques has capability to increase the effectiveness of an intervention [27, 50], and 2) cover techniques related to the different elements of behavior presented in the Behaviour Change Wheel [44]. The application of each behavior change technique is planned by using the Self-Determination Theory [23]. This means that when applying any of the selected BCTs it was reflected whether it supported (or hindered) autonomy, competence and relatedness and more internalized forms of motivation. For example, the feedback that the application provided included reflective questions, which, based on Silva et al. [17], we considered to support both autonomy and competence instead of icons or other kinds of trophies, which could lead to externalized motivation. The development process of the intervention’s contents and theoretical framework, as well as the counseling and tailoring will be described in more detail in an additional paper [34].

Another background theory for developing the intervention is trans-theoretical model (TTM) of change [46]. The original five stages of change in TTM (Precontemplation, Contemplation, Preparation, Action and Maintenance) were simplified into three for two reasons. First, as people in precontemplation stage would not engage, by the definition of the stage, in any activity towards behavior change, the application could not reach them. Second, in order to make the user interface of the application as user friendly as possible, the amount of available options should be minimized. Thus, the application contained three stages, Think and plan (containing contemplation and reparation stages), Act and do (action) and Maintain good (Maintenance). After receiving the topic related health information, the user could choose which stage they wanted and the application then provided goals and task in accordance to the stage.

The basis for the design of the mobile information system application is the Persuasive Systems Design (PSD) model [40], which stipulates the process involved in establishing effective software features that best match the needs and goals of the information system’s target users. Prior to the present application, the model has been used successfully in other health intervention designs and in research evaluating persuasive systems in the health domain (e.g. [37, 51–54]). It emphasizes the importance of analyzing the contexts of use, user and technology (that is, the persuasion context). Based on these, the development of a system proceeds to selecting the most feasible and effective persuasive features as the system requirements. The persuasive software features fall into four categories: Primary task support, Computer-human dialogue support, System Credibility support, and Social support. Each of these categories contains a number of established persuasion features. The transparency of the development process, as assumed in the PSD model, highlights the principle of excluding all aspects of coercion and deception when developing a BCSS.

In the present application, analysis of the persuasion context identified a need for goal-setting at the core of the application, and as such, goal- and task-setting are present in all parts of the application. Moreover, common features throughout the application modules include various features from the primary task support category, such as self-monitoring and virtual rehearsal, aiming for direct task-related support for the system users. All modules also include the computer-human dialogue features of praise as positive feedback, and provide suggestions for the user (tips and hints). In addition, modules are used that aim to promote immediate compliance with good health activities (namely, balancing work and free time, and breaking sedentary working habits) through reminders. As social support, the application uses the features of social comparison. Attention will also be paid to making the users feel that the information system application is credible.

The application is developed and programmed specifically for this intervention. The user will choose the module(s) freely as per the SDT, although one will be suggested by the system based on questions at the beginning of usage and another module will be suggested later on (based on social comparison). The contents of the application are divided into seven modules, which include total of 51 tasks, 11 videos e.g. mindfulness exercise, and seven self-monitoring tools e.g. pedometer. The user will receive information and tips about improving personal health and will choose tasks or will use self-monitoring tool of one’s own liking that will help with achieving the target behavior for example via self-reflection.

#### Primary, secondary and other outcomes

The primary outcome is the change from baseline in current Perceived Work Ability (PWA) in six months and secondary outcome the change from baselin in the Need for Recovery (NFR) scale in 6 months. The participants will evaluate their current PWA on a scale of 0 to 10, ten indicating lifetime best work ability. The question used for this is the first item of the Work Ability Index [55–57].

The NFR comprises 11 items [58]. Items include: “I find it difficult to relax at the end of a working day.” and “By the end of the working day, I feel really worn out.” The response scale is from 1 (never) to 4 (always). The NFR scale can be used to measure (early indications of) fatigue at work. Its score is calculated by adding up the individual’s scores on the 11 (recoded) items. This scale score is transformed into a scale ranging from 0 to 100. Higher scores indicate a higher degree of need for recovery after work [59, 60].

As background information, we will collect information on the entrepreneur (gender, age, place of residence, perceived health status, weight, height, education, years as entrepreneur) and enterprise (field of business, operational years, number of employees, existence of business plan, budget, turnover, arrangement of occupational health services).

#### Sample size

The primary outcome is perceived work ability (PWA). Barene et al. [61] found a score of 7.3 (SD 1.3) for the intervention group and 7.8 (SD 1.1) for control group. We aim to detect a 7% improvement in the perceived work ability score between the intervention and control group after 6 months of follow-up. Based on power of 0.8, and 2-sided alpha of 0.05, sample size of 124 + 124 microentrepreneurs is needed; 248 in total.

Based on our previous experiences of recruiting entrepreneurs for developmental projects, we estimate that less than 1% of those invited will be willing and able to participate. Thus, in order to get enough participants, we invite markedly more.

There are two main bases for specifying the target difference: a difference considered to be ‘important’ (for example, by a stakeholder group such as health professionals or patients), and a ‘realistic difference’ based upon current evidence (for example, seeking the best available estimates in the literature through some form of knowledge synthesis).

#### Recruitment of participants

The main recruitment route is an email invitation to microentrepreneurs. The source for email addresses is a Finnish commercial enterprise data register, called Bisnode. We use the Bisnode register because the register provides access to contacts with email addresses. It contains the contact information of over 468,000 organizations (0–9 employees) in continental Finland (data checked on 20-04-2017). After the exclusion of public and third sector organizations, housing cooperatives, and property companies and microenterprises without email address, the final total register base is 74,971 companies. The microentrepreneurs whose email-addresses are available will be sent an invitation letter via email. We expect 740 registrations for the study through this channel.

A media campaign was run from November 2017 to January 2018 in order to obtain a sufficient participation rate. The media campaign enabled those who were interested in participating in the study to register for the study on the FIOH website [62].

The aims of the media campaign were to make entrepreneurs aware of the topic of recovery from work, create willingness to change their habits toward better work ability, and finally to register for the study. The campaign included participating in events relevant for entrepreneurs, distributing printed handouts and flyers, a nationwide radio campaign (two weeks), press releases, articles and advertisements in newspapers, videos and posters on social media, targeted social media advertising, and blog texts. Both the researchers’ and research groups’ own social media outlets and the interest groups’ outlets were utilized to distribute the contents.

We used SDT principles (such as autonomy and relatedness) and different behavior change methods in designing the media campaign [63]. For instance, in videos and posters we used a modeling method in which real entrepreneurs presented their own examples of recovery from work. The aim was to present realistic, identifiable social role models and thereby create positive social pressure for other entrepreneurs to act in the same way, and boost their self-efficacy (i.e. belief that they are capable of acting in ways that enhance their recovery from work). By showing that other entrepreneurs do things to gain better recovery from work, and by giving concrete examples of such actions, the campaign aimed to provide information on social norms (others’ approval) and provide social support. What is more, rather than giving exact instructions, we supported autonomy through thought-provoking posters aiming to evoke reflection on one’s own emotions and behavior (compare with [53, 63–65]). The elaboration likelihood model (e.g. [66]) was also utilized in designing the messages for the radio advertisements. These messages were intended to be relevant for entrepreneurs and associated with their experiences, and to include an element of surprise, so that the entrepreneur would process them more carefully (central route processing). The messages were repeated to ensure better recollection. They presented arguments that turn common beliefs around (e.g. “breaks are not for lazy people”). The radio channels and playing times of the advertisements were chosen to reach particularly middle-aged male entrepreneurs, as it is estimated that they are especially less active than other entrepreneurs in participating in studies.

The two groups (self-registered and invited) will be randomized and analyzed separately because possible differences in the level of motivation might cause selection bias in the results. The entrepreneurs will be sent basic information about the study and a web-based questionnaire. When they return the completed questionnaire, they will be enrolled on a list. The order of the enrolment list is the basis for randomization into intervention or control groups.

A statistician did randomization using a table of random numbers (in batches of ten) and used SPSS for random sampling and allocation [67]). The participants who return the baseline survey, are randomized into blocks of ten and into either the intervention (IG) or the control group (CG) in order of response. The respondents invited through a company register and those who signed up voluntarily are randomized separately to ensure that an equal proportion of respondents obtained through both recruitment channels end up in both groups.

After randomization, the team responsible for the survey data management will upload the name, gender and email addresses of the intervention group through a secure channel to the team responsible for the application. Name is needed for personalization of the mobile app for the user, for a simple example it says hello ‘name’. Gender is used for gender specific feedback information in the app. Email is used for informing the intervention group that they belong to the intervention group and will be invited to download the application from the Google Play store. The email message will contain a personal account name, password and instructions for downloading and installing the application. In any technical issues, the IG can contact the application-specific helpdesk through email. At the same time, the project coordinator will send the CG an email message informing them that they belong to the wait list control group, which means that they do not miss out on anything but only receive the same intervention later. Whilst waiting they will be requested to fill in the follow-up surveys. Both survey data management and application teams will delete all identity information of study subjects right after the data collection has ended. All researchers will receive unidentified data to be used in data analyses. Only principal investigator of the consortium has access to identification data.

The pilot version of the application was distributed via a registered domain (website) to the pilot users, which involved them checking and altering their smartphone settings accordingly, as well as manually downloading and installing the application. We chose the Google Play store as the distribution platform during the intervention on the basis of feedback from the pilot users, in order to improve the ease of downloading and installing the application. This may require a few minor changes to the application to ensure that it could be published (and distributed to the users) via the Google Play store.

### Data collection, management and analysis

#### Measurements

**Fig. 2:**
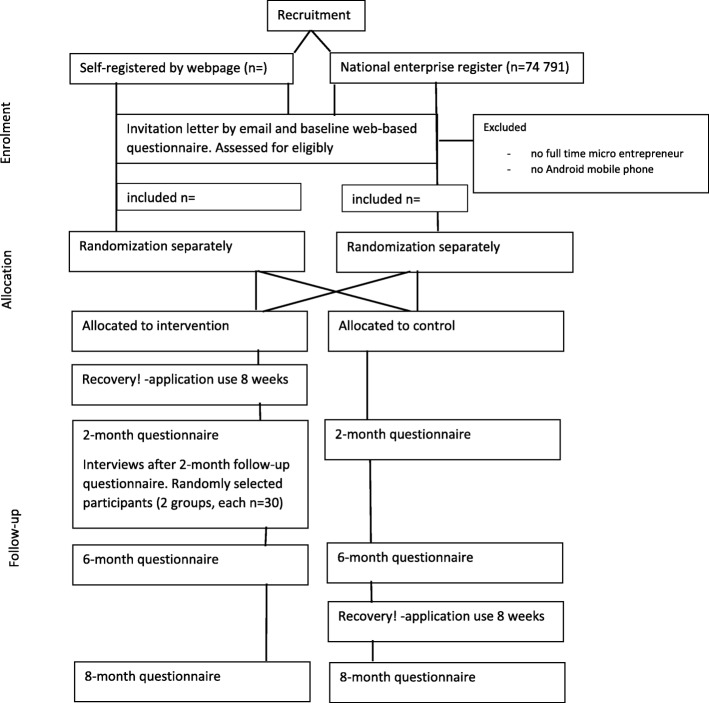
Participant flow chart and timeline

We will collect data via self-administered web-based questionnaires, the application and interviews (Fig. 2).

The self-administered web-based questionnaires at baseline and after two and six months from the beginning of the intervention will include questions measuring primary and secondary outcomes and other variables, and background information and questions whose responses will be used in evaluating the receipt and acceptability of the intervention and the users’ evaluations. Two reminders will be sent. Web-based surveys are used to promote data quality. Two months from the beginning of the intervention we will collect the experiences of and information on the use of the mobile information system application. The number of questions will be kept as minimal as possible in questionnaires, and the motivation will be enhanced in the invitation letter to obtain maximal participation rate.

The application will collect log-data for process evaluation purposes during the eight-week intervention. These data will be utilized to evaluate the usage of the application, for example through login and logout times, the activities completed, time spent on studying each content area as well as answering questions related to it. Only prespecified log data will be collected and stored on a secure server site.

Two randomly selected groups (adjusted for gender and route of enrolment for the study) of microentrepreneurs (*n* = 20–30 in both groups) will be interviewed after the usage period (two months) of the intervention. The participants will be asked to give written or taped informed consent according to the declaration of Helsinki. The first group will be interviewed about the user experience and usage of the mobile application according to themes such as persuasion, credibility and behavior change. The themes and questions have been tested by interviewing two pilot users. On the basis of lessons learned from the pilot, mainly the difficulty in recruiting interviewees, we included the option of confirming potential interest in interviews and of filling in more detailed contact information (phone number) to the baseline survey. The second group will be interviewed about themes related to experiences of work as an entrepreneur, the history and future hopes of the enterprise, professional identity, taking care of one’s well-being, recovery from work and work ability, lifestyle habits and the meaning of health, the effects of entrepreneurship on work ability and work recovery, the effects of work ability on being an entrepreneur and on business or work performance and productivity, the support needed for taking care of one’s own work ability, occupational health services, experiences of the use of the mobile application for promoting work recovery and work ability, and effects on lifestyle habits. The themes and questions will be tested in five interviews.

Data collected and created in the intervention trial of this project will be stored in centralized data servers. The data consist of raw data from different research technologies, pre-processed data from web-based inquiries, and data analysis results. Centralized processing and storage of the data enables efficient curation, harmonization and integration of the data, resulting in reliable high-quality research data. The data will be version controlled and backed up, ensuring its efficient storage and re-use. It will be accessible by all project partners, enabling secure data sharing and dissemination.

### Assessment of harm

This is a low risk intervention with only a few expected adverse events during the study. These may relate to the extra time needed to use the mobile application, problematic experiences with the technology, negative feelings about one’s own lifestyle habits and the need for change, and insufficient support and negative attitudes from one’s own social network towards participation. Sometimes lifestyle changes initially imply harmless yet negative consequences, such as muscle stiffness due to increasing physical activity. The use of the application, including counseling for healthier lifestyle habits, can also lead to non-expected positive effects to participants’ health and well-being. For example, family members may join the participant in physical exercise, which increases social cohesion in the family. Negative side effects e.g. disappointment and deficient treatment, drop-out are to be examined and we use both quantitative and qualitative research that will be able to detect the possible deterioration as well as the subjective experiences of the participants [62].

### Statistical analyses

The statistical differences in primary outcome measure between the intervention and control groups, will be measured using tests comparing the change from baseline in outcome to 6 months (for example T-test). The analyses will be performed using SPSS (version 24, SPSS Inc., Chicago, IL, USA), an alpha level of 0.05 being accepted as significant. Analyses will be controlled for covariates such as gender, age, education and group (self-registered or invited).

### Qualitative data analysis

The qualitative data from interviews will be transcribed and anonymized, and analyzed by content analysis [68]. At least two researchers will scrutinize the qualitative data and participate in their interpretation.

### Process evaluation

In order to examine the mechanism of change, we will conduct process evaluation. We will describe and analyze the recruitment process more carefully in separate process evaluation [69] papers. The characteristics of the invited participants and non-participants in this intervention study will be analyzed and compared to gain knowledge on which factors could be taken into account when developing new ways of promoting the work ability of microentrepreneurs. We also aim to analyze the implementation of the intervention (the dose and fidelity of the counseling delivered by the mobile application) and adherence to it. We will describe how the context related to microentrepreneurs may influence participation, the implementation of the intervention, or study outcomes, and how this has been taken into account in the intervention development process.

The evaluation will focus on the following components [70–72]:

1. **Context** For example, how the context of microentrepreneurship in Finland influenced the planning and development stages of the intervention, and how the context was taken into consideration.
2. **Reach** For example, how many of the contacted microentrepreneurs participated; how many of participants dropped out or were engaged in the study; who were they (subgroups?); what were the key reasons for engaging or not engaging in the intervention?
3. **Dose** For example, how and to what extent did the participants use the application and perform other intervention tasks (e.g. achieve changes in their lifestyles); what kind of actions did they perform; did contextual factors “outside the application” have an influence on the dose; did the subgroups differ in their interaction with the intervention/use of the application?
4. **Fidelity** For example, to what extent was the intervention delivered as intended and what factors influenced this? Description of the intervention (e.g. logic model), including its underlying theory and mechanisms of impact will be evaluated for understanding the extent to which the intervention and application were consistent with the underlying theory; whether participants followed the guidelines and suggestions; whether the application worked as intended.
5. **Implementation** For example, to what extent were the intervention and application implemented and received; to what extent were the different materials/activities/contents of application used/utilized; what kind of practical barriers did participants face during the process in terms of application and lifestyle change?
6. **Recruitment** For example, how was recruitment carried out in practice; what kind of participants engaged in the intervention; did they represent the target group in general; were some groups of entrepreneurs overrepresented/underrepresented; what were the barriers to recruiting participants?
7. **Participants’ experiences** of the intervention process and application (e.g. level of satisfaction, perceived usefulness) will be collected and analyzed, for example, how easy/difficult was it to follow intervention procedures and to use the application; what kind of experience was it; what were the key strengths and weaknesses of the intervention in general and of the application in particular; how could the intervention/application be developed; did the subgroups differ in regards to how they experienced the intervention?

The data collection methods for answering the above-mentioned questions will be web analytics, quantitative measures/scales integrated into pre-, post- and follow-up questionnaires and one-to-one interviews. We will use participant interviews and the results of short web-based questionnaires to evaluate the reception and acceptability of the intervention and the evaluations and interpretations of the causal mechanism of change.

## Discussion

This study aims to evaluate whether an occupational health promotion intervention using a mobile application is an effective way of reaching and promoting work ability and recovery from work among microentrepreneurs. Promoting the recovery from work and work ability of microentrepreneurs requires understanding the target group and the context in which they operate. They work long hours, have challenging timetables and difficulties in combining family and work. Lack of time is often suggested as the reason for not taking care of one’s health and work ability. Thus, a native mobile application may be suitable in this context.

Microentrepreneurs are an under-researched population in terms of recovery from work and work ability [9, 10, 42, 73], and thus this intervention study will be unique and provide important information that can be used in developing services for and studying microentrepreneurs. This study adapts an intervention to a very specific target group which could be a fruitful route to enlarge the reach of health promotion interventions compared to one-fits-all strategies. Secondly, it improves the knowledge on ways to enhance work ability of microentrepreneurs, whose companies form the vast majority of all companies in EU and thus are important for the economy and employment status in European Union. Thirdly, it combines both psychological and physiological ways/components to enhance recovery from work and promote work ability among microentrepreneurs. Further, forthcoming research work related to process evaluation will increase knowledge on the target group and its specific features to perform more effective interventions to promote work ability of microentrepreneurs.

Web-based and mobile applications usually share similar features, although they have different implementations when compared to each other. Most important difference is that native mobile applications can function without an internet connection, unlike web-based apps [43]. As regarding digital intervention research, web-based and mobile applications do not necessary have to be compartmentalized into different branches, although one should know the differences between them. In the area of digital and internet-based health interventions, mobile applications form one group of delivery systems. A review of digital mental health interventions in the workplace [74] identified interventions delivered as web-based systems, computer applications, e-mail, and as stand-alone computers. More generally in the health domain, the intervention delivery channels also include mobile applications [75]. The unique aspects of this study are that it will assess the effects of a mobile application utilizing a sound evidence base from the SDT and matching behavior change techniques, as well as the principles of the PSD model for designing and evaluating information systems through technological persuasion, without deception or coercion. Understanding the role of motivation (SDT) is expected to be essential when designing a mobile application for microentrepreneurs.

As far as we know this study is a starting point of a new research line. While earlier research on this topic in this target group (microentrepreneurs) does not exist, process evaluation might even be the most valuable research aim in order to improve the feasibility of the forthcoming studies in this topic among this target group. Process evaluation of the intervention will provide deeper knowledge on the factors related to the context, reach and recruitment and fidelity and dose, which all are important factors related to the feasibility and effectiveness of the intervention study among microentrepreneurs. Because the microentrepreneurs include very heterogenous group of entrepreneurs, the context is essential, and it might be difficult to develop a feasible and suitable intervention, although it is done by cocreation. Fidelity and dose might be very different among microentrepreneurs compared to those among wage earners. Heterogeneity of the target group may hamper the reach and recruitment, and thus to invite microentrepreneurs largely, not only some specific subgroup of them might also be a limitation of the study.

Further, we do not know if our hypothesis related to the main components of the intervention are suitable and good for microentrepreneurs, because of the lack and research gap of earlier research. We will use mixed methods to collect data, we will have a good possibility to learn more about this target group and about the most important issues related to the promotion of work ability by the means we will use in this intervention study. This gap of intervention studies among microentrepreneurs is surprising, because microentrepreneurs are important from the societal point of view: microenterprises cover about 93% of all companies in European Union, and thus are very important for the economy and employment of EU [1, 2, 8]. Microenterprises are the key persons of these companies with less than 10 employees, and their work ability might be the basis for the profitability and success of their business.

There can be factors, that can confound the effects of the intervention, such as economic situation or smartphone’s operational environment (we chose Android) and its technical problems and misfunctioning. Further, we try to take into account seasonal issues, e.g. holidays, but it can be that microentrepreneurs have their holidays in different season that wage earners usually have, or have no or shorter holidays.

This innovative approach, if successful, may provide a means through which to deliver a low-cost work ability promotion program that has the potential to reach large groups, particularly among microentrepreneurs, which form the vast majority of all enterprises and do not usually have occupational health services. A novel aspect of this project is the development of a new kind of mobile application, based on strict science, and specially designed for microentrepreneurs.

## Data Availability

Not applicable – this is a research protocol and does not contain any data. Future data will be available from the corresponding author on reasonable request.

## Abbreviations

BCSS: The Behaviour Change Support Systems
CG: Control group
FIOH: Finnish Institute of Occupational Health
IG: Intervention group
NfR: Need for Recovery
PSD: The Persuasive Systems Design (PSD) model
PWA: Perceived Work Ability
SDT: Self Determination Theory
